# The effect of apolipoprotein E polymorphism on serum metabolome – a population-based 10-year follow-up study

**DOI:** 10.1038/s41598-018-36450-9

**Published:** 2019-01-24

**Authors:** Juho-Pekka Karjalainen, Nina Mononen, Nina Hutri-Kähönen, Miikael Lehtimäki, Markus Juonala, Mika Ala-Korpela, Mika Kähönen, Olli Raitakari, Terho Lehtimäki

**Affiliations:** 1 Department of Clinical Chemistry, Fimlab Laboratories and Finnish Cardiovascular Research Center-Tampere, Faculty of Medicine and Life Sciences, University of Tampere, Tampere, Finland; 2 Department of Pediatrics, Tampere University Hospital and Faculty of Medicine and Life Sciences, University of Tampere, Tampere, Finland; 3 Department of Medicine, University of Turku, and Division of Medicine, Turku University Hospital, Turku, Finland, Murdoch Children’s Research Institute, Melbourne, Victoria, Australia; 4 Computational Medicine, Faculty of Medicine, University of Oulu and Biocenter Oulu, Oulu, Finland; 5 NMR Metabolomics Laboratory, School of Pharmacy, University of Eastern Finland, Kuopio, Finland; 6 Medical Research Council Integrative Epidemiology Unit at the University of Bristol, Bristol, UK; 7 Population Health Science, Bristol Medical School, University of Bristol, Bristol, UK; 8 Systems Epidemiology, Baker Heart and Diabetes Institute, Melbourne, VIC, Australia; 9 Department of Epidemiology and Preventive Medicine, School of Public Health and Preventive Medicine, Faculty of Medicine, Nursing and Health Sciences, The Alfred Hospital, Monash University, Melbourne, VIC, Australia; 10 Department of Clinical Physiology, Tampere University Hospital, and Finnish Cardiovascular Research Center - Tampere, Faculty of Medicine and Life Sciences, University of Tampere, Tampere, Finland; 11 Department of Clinical Physiology and Nuclear Medicine, Turku University Hospital, and Research Centre of Applied and Preventive Cardiovascular Medicine, University of Turku, Turku, Finland

## Abstract

Apolipoprotein E (apoE) is the key regulator of plasma lipids, mediating altered functionalities in lipoprotein metabolism – affecting the risk of coronary artery (CAD) and Alzheimer’s diseases, as well as longevity. Searching pathways influenced by apoE prior to adverse manifestations, we utilized a metabolome dataset of 228 nuclear-magnetic-resonance-measured serum parameters with a 10-year follow-up from the population-based Young Finns Study cohort of 2,234 apoE-genotyped (rs7412, rs429358) adults, aged 24–39 at baseline. At the end of our follow-up, by limiting FDR-corrected p < 0.05, regression analyses revealed 180/228 apoE-polymorphism-related associations with the studied metabolites, in all subjects – without indications of apoE *x* sex interactions. Across all measured apoE- and apoB-containing lipoproteins, ε4 allele had consistently atherogenic and ε2 protective effect on particle concentrations of free/esterified cholesterol, triglycerides, phospholipids and total lipids. As novel findings, ε4 associated with glycoprotein acetyls, LDL-diameter and isoleucine – all reported biomarkers of CAD-risk, inflammation, diabetes and total mortality. ApoE-subgroup differences persisted through our 10-year follow-up, although some variation of individual metabolite levels was noticed. In conclusion, apoE polymorphism associate with a complex metabolic change, including aberrations in multiple novel biomarkers related to elevated cardiometabolic and all-cause mortality risk, extending our understanding about the role of apoE in health and disease.

## Introduction

Coronary artery disease (CAD) along with carotid artery disease and consequent strokes are the leading causes of death worldwide – according to the latest statistics ca. 15 million people died of these cardiovascular diseases (CVD) in 2016^1^. In addition, as populations around the world are rapidly ageing^2^, the importance of paying attention to the causes of atherosclerotic plaque formation and its clinical consequences are also increasing. It has been shown that genetic components affect the atherosclerosis and CAD risk^3,4^. However, the genetic or epigenetic mechanisms targeted for improved prevention and treatment by personalized medicine are not yet known well enough.

Apolipoprotein E (apoE) is a glycoprotein consisting of 299 amino acids, and it functions as the key regulator of plasma lipid levels. Two single-nucleotide polymorphisms (SNPs, rs429358 and rs7412) at locus 19q13.31 determine three common allelic variants of the apoE gene: ε2, ε3 and ε4. Respectively, these alleles code three protein isoforms: E2, E3 and E4. Of the six apoE genotypes, ε2/2, ε3/2, ε3/3, ε4/2, ε4/3 and ε4/4, the homozygote ε3/3 is the most prevalent, so called reference (or parent) genotype, and the E3 isoform of the protein is associated with normal (i.e., average) plasma lipid levels. Compared with E3, E2 and E4 isoforms have been discovered to have altered functionalities in promoting clearance of triglyceride (TG) rich lipoproteins from plasma^5,6^. The reported prevalence of different alleles within human population is by average: ε2/7%, ε3/78% and ε4/14%^6^. The associations of ε4 with elevated serum total cholesterol and LDL (low-density lipoprotein) cholesterol (LDL-C) values have been recognized for a long time^7^ and the allele ε4 has been linked to the risk of atherosclerosis^8^ and CAD, whereas ε2 has been shown to e.g., reduce carotid artery intima-media thickness and coronary artery calcification^9,10^. Nevertheless, there are also further presented hypotheses for the effects of apoE polymorphism on the development and severity of CAD and other diseases^5,6,11,12^. For example, apoE also regulates lipid transport and cholesterol homeostasis in the brain and the ε4 allele has been linked to Alzheimer’s disease^6,11^ – another common killer among an ageing population. Finnish population has higher than average incidence of atherosclerosis and the nationwide prevalence of ε4 has been reported to be about 20%^7,13^.

While the causality of high serum total cholesterol and LDL-C with CAD is a generally recognized feature, LDL-C itself may not be sensitive or specific enough for predicting atherogenesis^14^. For example, LDL-TG has been recommended as a more targeted parameter^15^, whereas a recent systematic review suggests several novel CAD risk markers found by state-of-art lipidomics and metabolomics methods^14^. On the other hand, the significance of apoE polymorphism on the susceptibility and development of CAD is also debated – it has been investigated in several different studies, both epidemiologically and clinically, often with controversial results^16–19^. In conclusion, further research is needed on clarifying the metabolic pathways and mechanisms of the pathogenesis of CAD – as well as on the role of genetic control, including apoE.

Nevertheless, an autopsy study of middle-aged Finnish men has indicated that apoE polymorphisms associate with the area of total atherosclerotic lesions in age- and tissue-dependent manner^8^. On the other hand, studies with relatively young Finnish subjects have not found evidence of apoE polymorphism being an independent genetic determinant of early atherosclerosis signs - carotid artery compliance (CAC), carotid artery intima media thickness (IMT) and brachial artery flow-mediated dilation (FMD)^20,21^. However, polymorphisms of apoE, as well as its promoter have been recognized to associate with standard lipid and lipoprotein profile changes of young and middle-aged Finns^20,22,23^.

Worldwide, despite numerous apoE-related association studies for standard serum lipid profiles^24^, much-needed information is clearly lacking at more detailed and thorough serum metabolome level. The serum metabolite profile can be defined significantly more comprehensively and accurately with high-throughput serum nuclear magnetic resonance (NMR) spectroscopy^25^, which has been used in the cohort profiling of our present study. With more thorough quantification combined with our systematically followed, large cohort we aim to clarify apoE functionalities linked to metabolic pathways of atherogenesis and regulation of longevity – thus extending our current understanding about the role of apoE in health and disease.

## Materials and Methods

### Study population and data sources

The Cardiovascular Risk in Young Finns Study (YFS) is a Finnish longitudinal general population study on the evolution of cardiovascular risk factors from childhood to adulthood^26^. The study began in 1980, when 3,596 children and adolescents aged 3–18 years were randomly selected from five university hospital catchment areas in Finland. In 2001, 2,288 participants aged 24–39 years attended the 21-year follow-up. 2,200 participated in the 27-year follow-up in 2007, and 2,063 contributed to the 31-year follow-up in 2011. Of these subjects, we included those for whom the apoE genotype data and at least 80% of the NMR-measured metabolic parameters were available. Therefore, 2,234 participants (2001), 2,148 participants (2007) and 1,918 participants (2011) contributed to the cross-sectional association analyses of apoE genotype and serum metabolic profile. In the longitudinal analyses for comparing the metabolic level differences between two follow-up measurements, we included the subjects who attended both the follow-ups in question – 1,751 participants (2001–2007), 1,734 participants (2007–2011) and 1,647 participants (2001–2011). In two-way repeated-measurement analyses of variances, we included 1,471 subjects who attended all the three consecutive follow-ups (2001, 2007, 2011). The YFS was approved by the 1^st^ ethical committee of the Hospital District of Southwest Finland and by local ethical committees (1^st^ Ethical Committee of the Hospital District of Southwest Finland, Regional Ethics Committee of the Expert Responsibility area of Tampere University Hospital, Helsinki University Hospital Ethical Committee of Medicine, The Research Ethics Committee of the Northern Savo Hospital District and Ethics Committee of the Northern Ostrobothnia Hospital District). The study protocol of each study phase corresponded to the proposal by the World Health Organization. All present subjects gave written informed consent and the study was conducted in accordance with the Helsinki declaration. At prior follow-ups of YFS, informed consent of every participant under the age of 18 was obtained from a parent and/or legal guardian.

### Clinical and biochemical measurements and their use in statistical standardization

To eliminate effects of the most probable error sources, a comprehensive set of clinical background information was analysed as confounding candidates. The effect of BMI (measured weight [kg]/measured height squared [m²]) was considered by including it in the final regression models as a covariate. Based on questionnaires, daily smoking (yes/no), hormonal birth control of women (yes/no), cholesterol lowering medication (yes/no) and socio-economic status based on occupation (manual/lower non-manual/upper non-manual) were all tested as covariates to associate with several different metabolic levels, but to have negligible or zero effect on the apoE β-values. The distributions of alcohol consumption (daily portions based on a questionnaire, one portion equalling 12 g of pure alcohol) and physical activity index (graded 5–15, the higher the value the more physically active, evaluation method described elsewhere^27^) were well comparable in every analysed apoE subgroup and therefore not confounding. Also, there were only a few pregnant women and persons with diagnosed diabetes (evaluated with questionnaires) and their distributions did not differ in the analysed subgroups. Parameters describing the cardiovascular status, such as systolic blood pressure (defined as an average of three consecutive measurements with random-zero mercury sphygmomanometer), hypertension (based on a questionnaire for medically diagnosed hypertension, yes/no) and high-sensitivity CRP (hs-CRP, measured with an automatic analyser Olympus AU400) might well reflect variations of metabolic measures, i.e., could be considered more as consequent rather than confounding factors. Nevertheless, differences in these measures between the subgroups were also found to be minor – a significant majority of the study population being basically healthy. Based on the described background analyses and to avoid unnecessary selection error, additional exclusions were not made in the final analyses. The effect of possible information bias may well be neglected in our results, when comparing the subgroups to each other – bias (if any) being presumably distributed comparably.

### ApoE genotyping

ApoE alleles (ε2, ε3, ε4) were determined based on SNPs rs7412 and rs429358 haplotypes. Genomic DNA was extracted from peripheral blood leukocytes by using QIAamp DNA Blood Minikit and automated biorobot M48 extraction (Qiagen, Hilden, Germany). Genotyping was performed by using Taqman SNP Genotyping Assays (C__904973_10, C_3084793_20) and ABI Prism 7900 HT Sequence Detection System (Applied Biosystems, Foster City, CA, USA). As a quality control, water controls, random duplicates and known control samples were run in parallel with unknown DNA samples.

### Metabolic profiling

High-throughput NMR spectroscopy was used for the absolute quantification of serum metabolites. The metabolomics set includes 228 quantified metabolic parameters (detailed description given in a Supplementary Table S1) and covers multiple metabolic pathways, including lipoprotein lipids and subclasses, fatty acids and fatty acid compositions, as well as amino acids and glycolysis precursors. All molecular measures are quantified in a single experimental setup, constituting both established and novel metabolic risk factors. This NMR-based metabolite profiling has previously been used in various epidemiological and genetic studies^28^ and has been reviewed recently^25^. Details of the experimentation have been described elsewhere^25,29^. In addition, we used standard lipid panels (described in^30^) for comparing/verifying our metabolomics lipid results.

### Statistical methods

All statistical analyses were conducted with R program version >3.1.2 (https://www.r-project.org/) using PC. To facilitate comparisons across all metabolites, association magnitudes (β-values) are reported in scaled standard deviation (SD) fractions (units) of normalized, ln-transformed metabolite concentrations. In sex-stratified cross-sectional analyses, separate sex-specific scaling was applied to NMR measures.

Cross-sectional and longitudinal associations of apoE genotype and serum metabolic profile were analysed using linear multivariable regression models, with each metabolic measure (in the longitudinal analyses the difference of the corresponding metabolic measures) as the outcome and apoE genotype as the main explanatory value. All the regression models were adjusted for age and BMI, in the longitudinal analyses used models were adjusted also for BMI-change. The effect of sex was analysed in both ways with models stratified by sex, as well as models with sex as a covariate. Interaction of apoE and BMI (apoE × BMI, apoE × BMI-change) was tested to have negligible or zero effect on the apoE β-values and was therefore excluded from the final models. Prior to testing the interaction effects of BMI, measures of BMI and BMI-change were mean-centred. Similarly, interaction of apoE and sex (apoE × sex) was excluded from the final models for not showing an effect within any metabolite (tested p > 0.05). ApoE genotype was included in the model as a categorical variable. First, the overall effect of apoE genotype on the linear fit was F-tested with implementing the Benjamini-Hochberg procedure^31^ in false discovery rate (FDR) correction and setting the limit at p < 0.05. After that, apoE genotypes were post-hoc compared with each other with t-tests (implementing FDR correction), the values of which were inherited from the linear regression function (lm) in R. 95% confidence intervals were also calculated, allowing a better comparison of the apoE groups.

In two-way repeated-measures analyses of variances, isolated missing measures of random individuals were mean value replaced. As described above, the analysis was performed with ln-transformed and scaled (normalized) data. The independent variables (time and apoE genotype) were factored, and the dependent variable (metabolic measure) was a continuous variable. Main effects of time and apoE, as well as interaction effect (apoE × time) were analysed. Natural variation between participants was considered with an error term. There were no significant outliers present in the dataset.

## Results

### Characteristics of cross-sectional and longitudinal analyses

The summary of the descriptive data for the YFS study subjects at the baseline (2001) and the end (2011) of our follow-up is presented in Table 1, the frequencies of different apoE genotypes in the study population are shown in Table 2. More details (including follow-up data of year 2007) can be found in Supplementary Tables S2 and S3.

**Table 1:** Summary descriptive data for the YFS cohort in 2001 and 2011. Values are mean (SD) or n (%).

	2001			2011		
	All	Male	Female	All	Male	Female
Number of subjects	2234	1004 (44.9)	1230 (55.1)	1918	860 (44.8)	1058 (55.2)
Age [years]	31.7 (5.0)	31.7 (5.0)	31.7 (5.0)	41.9 (5.0)	41.9 (5.1)	41.9 (5.0)
BMI [kg/m²]	25.1 (4.4)	25.7 (4.1)	24.5 (4.6)	26.5 (5.0)	27.0 (4.3)	26.1 (5.5)
Daily smokers	533 (23.9)	296 (29.5)	237 (19.3)	263 (13.7)	133 (15.5)	130 (12.3)
Cholesterol lowering medicated	7 (0.3)	6 (0.6)	1 (0.1)	70 (3.6)	48 (5.6)	22 (2.1)
Diabetes mellitus type 2	1 (0.0)	1 (0.1)	0 (0.0)	69 (3.6)	32 (3.7)	37 (3.5)
Hypertension	40 (1.8)	22 (2.2)	18 (1.5)	160 (8.3)	81 (9.4)	79 (7.5)
Total cholesterol [mmol/L]	5.20 (1.12)	5.16 (1.06)	5.24 (1.17)	5.33 (1.07)	5.40 (1.12)	5.27 (1.02)
VLDL cholesterol [mmol/L]	0.81 (0.31)	0.90 (0.32)	0.75 (0.29)	0.76 (0.33)	0.88 (0.35)	0.67 (0.27)
IDL cholesterol [mmol/L]	0.83 (0.23)	0.84 (0.22)	0.82 (0.23)	0.86 (0.22)	0.88 (0.23)	0.83 (0.20)
LDL cholesterol [mmol/L]	1.94 (0.60)	2.00 (0.61)	1.89 (0.60)	2.05 (0.60)	2.17 (0.63)	1.96 (0.57)
HDL cholesterol [mmol/L]	1.78 (0.42)	1.43 (0.33)	1.78 (0.42)	1.65 (0.42)	1.46 (0.36)	1.81 (0.40)
Triglycerides [mmol/L]	1.29 (0.69)	1.45 (0.73)	1.17 (0.62)	1.31 (0.87)	1.57 (0.96)	1.10 (0.73)
apoA-I [g/L]	1.68 (0.27)	1.59 (0.21)	1.76 (0.28)	1.70 (0.24)	1.62 (0.21)	1.77 (0.25)
apoB [g/L]	0.98 (0.24)	1.03 (0.24)	0.94 (0.23)	1.00 (0.25)	1.08 (0.26)	0.93 (0.22)

Abbreviations: BMI, body mass index; VLDL, very-low-density lipoprotein; IDL, intermediate-density lipoprotein; LDL, low-density lipoprotein; HDL, high-density lipoprotein; apoA-I, Apolipoprotein A-I; apoB, Apolipoprotein B.

**Table 2:** Frequencies, n (%), of different apoE genotypes in the YFS cohort in 2001 and 2011.

	2001			2011		
	All	Male	Female	All	Male	Female
ε2/2	4 (0.2)	2 (0.2)	2 (0.2)	4 (0.2)	2 (0.2)	2 (0.2)
ε3/2	142 (6.4)	46 (4.6)	96 (7.8)	130 (6.8)	47 (5.5)	83 (7.8)
ε3/3	1280 (57.3)	585 (58.3)	695 (56.5)	1096 (57.1)	495 (57.6)	601 (56.8)
ε4/2	44 (2.0)	18 (1.8)	26 (2.1)	36 (1.9)	13 (1.5)	23 (2.2)
ε4/3	684 (30.6)	317 (31.6)	367 (29.8)	578 (30.1)	271 (31.5)	307 (29.0)
ε4/4	80 (3.6)	36 (3.6)	44 (3.6)	74 (3.9)	32 (3.7)	42 (4.0)
TOTAL	2234	1004	1230	1918	860	1058

### Cross-sectional associations of apoE genotypes within 228 tested serum metabolic parameters in YFS

**Figure 1:**
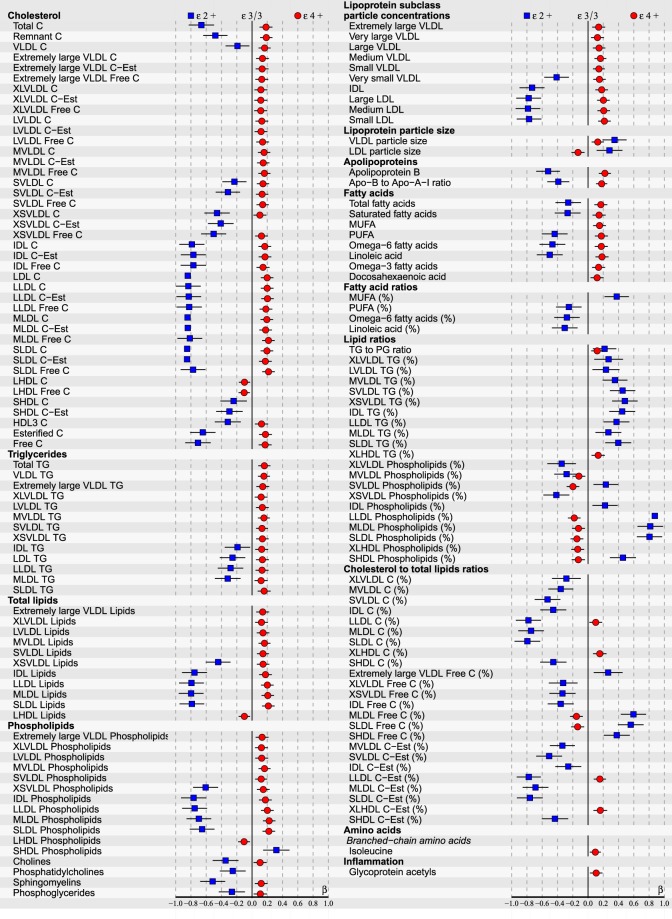
ApoE effects on 154/228 (p < 0.05 after false discovery rate correction) NMR-based serum metabolic measures in all subjects (n = 2234) of YFS cohort participated in 2001. Statistics: Regression models are adjusted for age, BMI and sex. Regression β-coefficients (x-axis) indicate in standard deviation (SD) units the change in metabolite level over apoE genotype subgroups (ε2+, ε3/3, ε4+). The most common ε3/3 subgroup (n = 1280) is set at the origin (zero SD) and post-hoc compared with ε2+ (squares) and ε4+ subgroups (circles). β-values with 95% CI are scaled to SD increments from normalized i.e., ln-transformed metabolic measures. For clarity of illustration, only the results of the analyses with p < 0.05 after false discovery rate correction are shown here. Definitions: apoE ε2+ subgroup (ε2/2, ε3/2 combined; n = 146) and apoE ε4+ subgroup (ε4/2, ε4/3, ε4/4 combined; n = 808).

**Figure 2:**
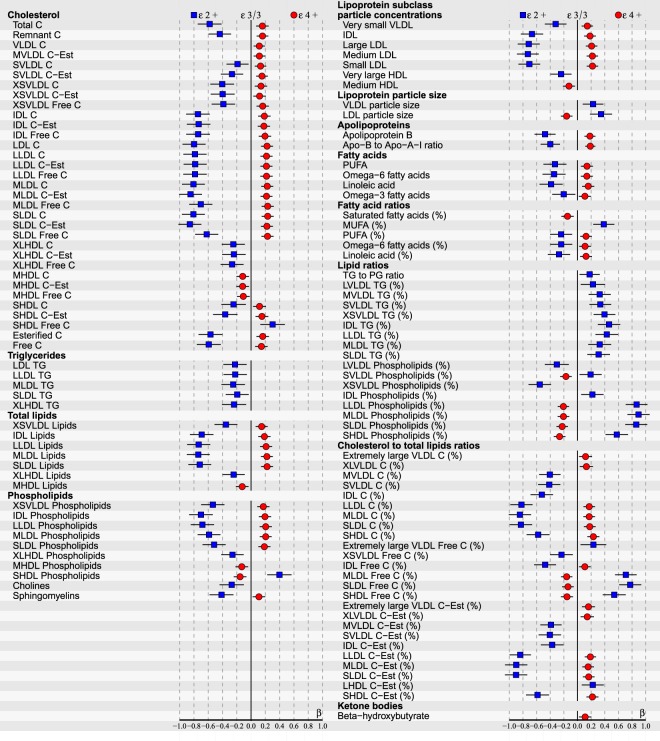
ApoE effects on 118/228 (p < 0.05 after false discovery rate correction) NMR-based serum metabolic measures in all subjects (n = 2148) of YFS cohort participated in 2007. Statistics: Regression models are adjusted for age, BMI and sex. Regression β-coefficients (x-axis) indicate in standard deviation (SD) units the change in metabolite level over apoE genotype subgroups (ε2+, ε3/3, ε4+). The most common ε3/3 subgroup (n = 1235) is set at the origin (zero SD) and post-hoc compared with ε2+ (squares) and ε4+ subgroups (circles). β-values with 95% CI are scaled to SD increments from normalized i.e., ln-transformed metabolic measures. For clarity of illustration, only the results of the analyses with p < 0.05 after false discovery rate correction are shown here. Definitions: apoE ε2+ subgroup (ε2/2, ε3/2 combined; n = 144) and apoE ε4+ subgroup (ε4/2, ε4/3, ε4/4 combined; n = 769).

**Figure 3:**
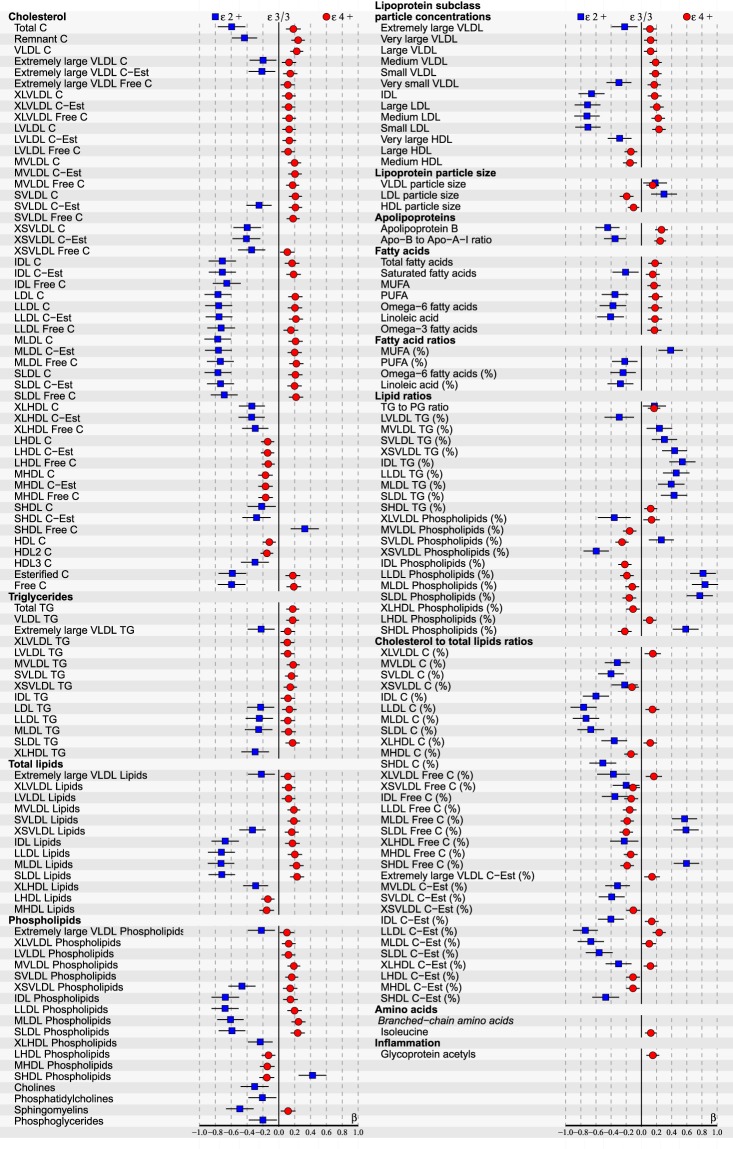
ApoE effects on 180/228 (p < 0.05 after false discovery rate correction) NMR-based serum metabolic measures in all subjects (n = 1918) of YFS cohort participated in 2011. Statistics: Regression models are adjusted for age, BMI and sex. Regression β-coefficients (x-axis) indicate in standard deviation (SD) units the change in metabolite level over apoE genotype subgroups (ε2+, ε3/3, ε4+). The most common ε3/3 subgroup (n = 1096) is set at the origin (zero SD) and post-hoc compared with ε2+ (squares) and ε4+ subgroups (circles). β-values with 95% CI are scaled to SD increments from normalized i.e., ln-transformed metabolic measures. For clarity of illustration, only the results of the analyses with p < 0.05 after false discovery rate correction are shown here. Definitions: apoE ε2+ subgroup (ε2/2, ε3/2 combined; n = 134) and apoE ε4+ subgroup (ε4/2, ε4/3, ε4/4 combined; n = 688).

Due to small subgroups of apoE ε2/2 and ε4/2, as well as ε4/4 in our population-based cohort (see Table 2), apoE genotypes were clustered into three larger subgroups for better statistical comparison: The reference group of ε3/3, as well as ε2+ (consisting of ε2/2 and ε3/2) and ε4+ (including all ε4 carriers: ε4/2, ε4/3 and ε4/4). The cross-sectional associations with p < 0.05 after FDR, including 95% CI, of apoE genotypes within the total of 228 tested serum metabolic parameters measured during the 10-year follow-up period are illustrated in Figs 1–3 and Supplementary Figures S4–6. For a more legible presentation, the statistics with higher p-values were excluded from these figures, but are provided in Supplementary datasets S7–9. The data of Figs 1–3 with exact p-values is also included in S7–9. Figures 1–3 represent the associations in all subjects, at baseline in 2001 (154/228 associations with FDR corrected p < 0.05), as well as in 2007 (118/228) and 2011 (180/228) follow-ups respectively. In Supplementary Figures 4–6 results obtained from the sex-stratified analyses are shown separately for men and women over the same period. Furthermore, the complete results from additional homozygote comparisons between ε3/3 and ε4/4 are provided in Supplementary datasets S10–12 – ε2/2 group was too small to be analysed separately.

At the end of the follow-up in 2011, by limiting FDR-corrected p < 0.05, multivariate regression analyses revealed a total of 180/228 metabolic associations with apoE polymorphisms in all subjects (Fig. 3). ApoE ε4 allele had a consistent atherogenic and ε2 protective effect across all measured apoE- (chylomicron remnants, very-low-density lipoproteins (VLDL), intermediate-density lipoproteins (IDL), high-density lipoproteins (HDL)) and apolipoprotein B (apoB) -containing (VLDL, IDL, LDL) lipoproteins with an influence on particle concentrations of free/esterified cholesterol, triglycerides, phospholipids and total lipid contents, as well as on LDL and VLDL particle sizes. The observed apoE subgroup differences remained relatively consistent during the entire 10-year follow-up, although some variation of individual metabolite levels (expressed in SDs from the reference group ε3/3) was noticed. There were no apoE × sex interactions found in relation to any studied metabolite (p > 0.05), during any of the three different timepoints measured, justifying the main analyses being performed with all subjects combined.

In the sex-stratified cross-sectional analyses performed for the three consecutive follow-ups (2001, 2007 and 2011), it is difficult to distinguish any clearly consistent, sex-specific differences in the associations of individual metabolic measures with apoE polymorphism. However, at least as an analytical outcome many of the differences between the compared apoE subgroups decrease with men and increase with women towards the 2011 analyses (as illustrated in Supplementary Figures S4–6) – especially notable in the apoE ε2+ subgroup. As isolated remarks on sex-stratified results, elevation of glycoprotein acetyls (GlycA) and isoleucine levels (with p < 0.05 after FDR) were found only in the apoE ε4+ group of men, in the 2011 follow-up (see Supplementary Figure S6).

Possible effects of interactions between the main term (apoE genotype) and the covariates (described in Materials and methods Section) in the multivariate linear regression model were all tested to have either zero or negligible weight on the cross-sectional association outcomes (apoE β-values). Therefore, interaction terms were excluded from the final models.

Our metabolomics lipid results were parallel/confirmed using standard lipid panels. Total cholesterol, LDL-C, as well as apoB were all elevated among the apoE ε4 allele carriers and lowered among the ε2 carriers as compared with the most common apoE ε3/3 group, in all follow-up points, as expected (data not shown here). In addition, at the end of our follow-up period in 2011 ε4 carriers expressed also elevated TG and lowered HDL-C – these two parameters did not differ statistically between the ε2 carriers and the ε3/3 group at any follow-up measurement.

### Longitudinal differences between apoE genotypes within 228 tested serum metabolic parameters in YFS

**Figure 4:**
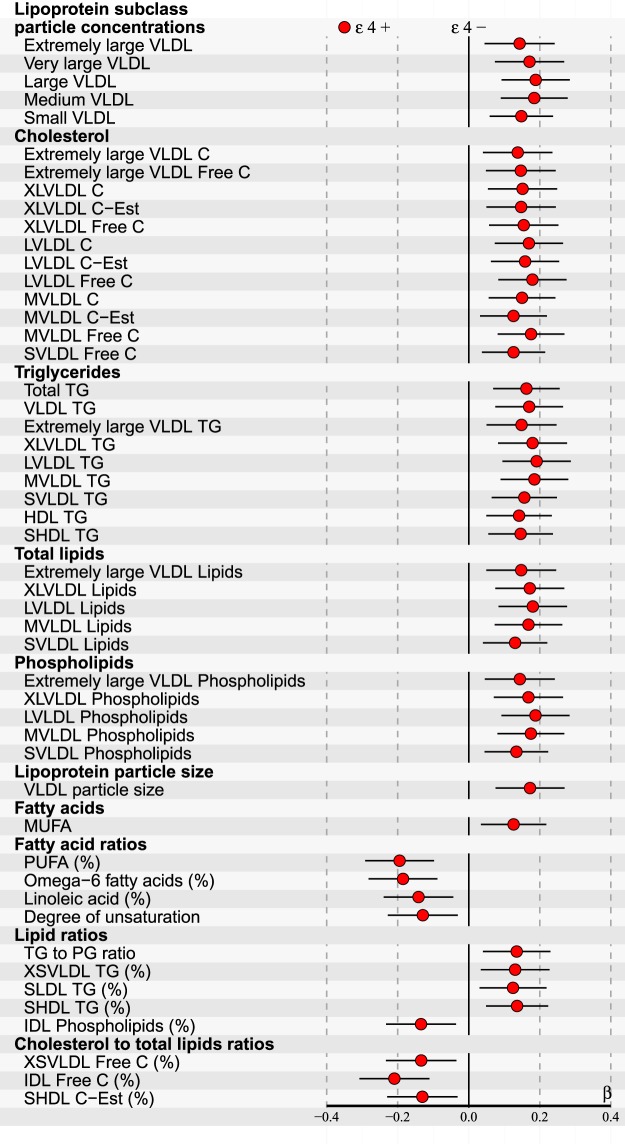
ApoE effects on the longitudinal changes (i.e., calculated difference between 2007–2011) of 44/228 (p < 0.05 after false discovery rate correction) NMR-based serum metabolic measures in all subjects participated in both follow-ups (n = 1734). Statistics: Regression models are adjusted for age, sex, baseline BMI and BMI-change. Regression β-coefficients (x-axis) indicate in standard deviation (SD) units the difference of metabolite level change between two apoE genotype subgroups (ε4− and ε4+). ε4− group is set at the origin (zero SD) and post-hoc compared with ε4+ group (circles). β-values with 95% CI are scaled to SD increments from normalized i.e., ln-transformed metabolic change measures. For clarity of illustration, only the results of the analyses with p < 0.05 after false discovery rate correction are shown here. Definitions: apoE ε4− subgroup (ε2/2, ε3/2, ε3/3 combined; n = 1112) and apoE ε4+ subgroup (ε4/2, ε4/3, ε4/4 combined; n = 622).

Longitudinal changes within 228 serum metabolic parameters and possible variation over apoE genotypes were investigated using calculated level differences between two follow-up measurements (i.e., 2001–2011, 2001–2007 and 2007–2011). For improved statistical power, apoE genotypes were clustered into two subgroups: The reference group of apoE ε4− (consisting of ε2/2, ε3/2 and ε3/3) and apoE ε4+ (including ε4/2, ε4/3 and ε4/4). For distinguishing any differences between the two compared subgroups, we discovered that it was essential to adjust the multivariate linear regression models also with the change of body mass index (BMI). Finally, 44/228 differences limited by p < 0.05 after FDR were found, however, only in the analyses of the follow-up interval of 2007–2011. These results are illustrated in Fig. 4. Allowing a more legible presentation, the statistics with higher p-values were excluded from this figure, but are provided in Supplementary Dataset S13. The data of Fig. 4 with exact p-values is also included in S13. Consistently increased differences of VLDL- and TG-related metabolites can be observed among the apoE ε4+ subgroup. The effects of main term (apoE genotype) and covariate (described in Materials and methods Section) interactions were tested to be of no relevance, and therefore the interaction terms were excluded from the final models.

### Novel associations of apoE polymorphism with previously identified biomarkers of CAD, inflammation, diabetes and all-cause mortality

**Figure 5:**
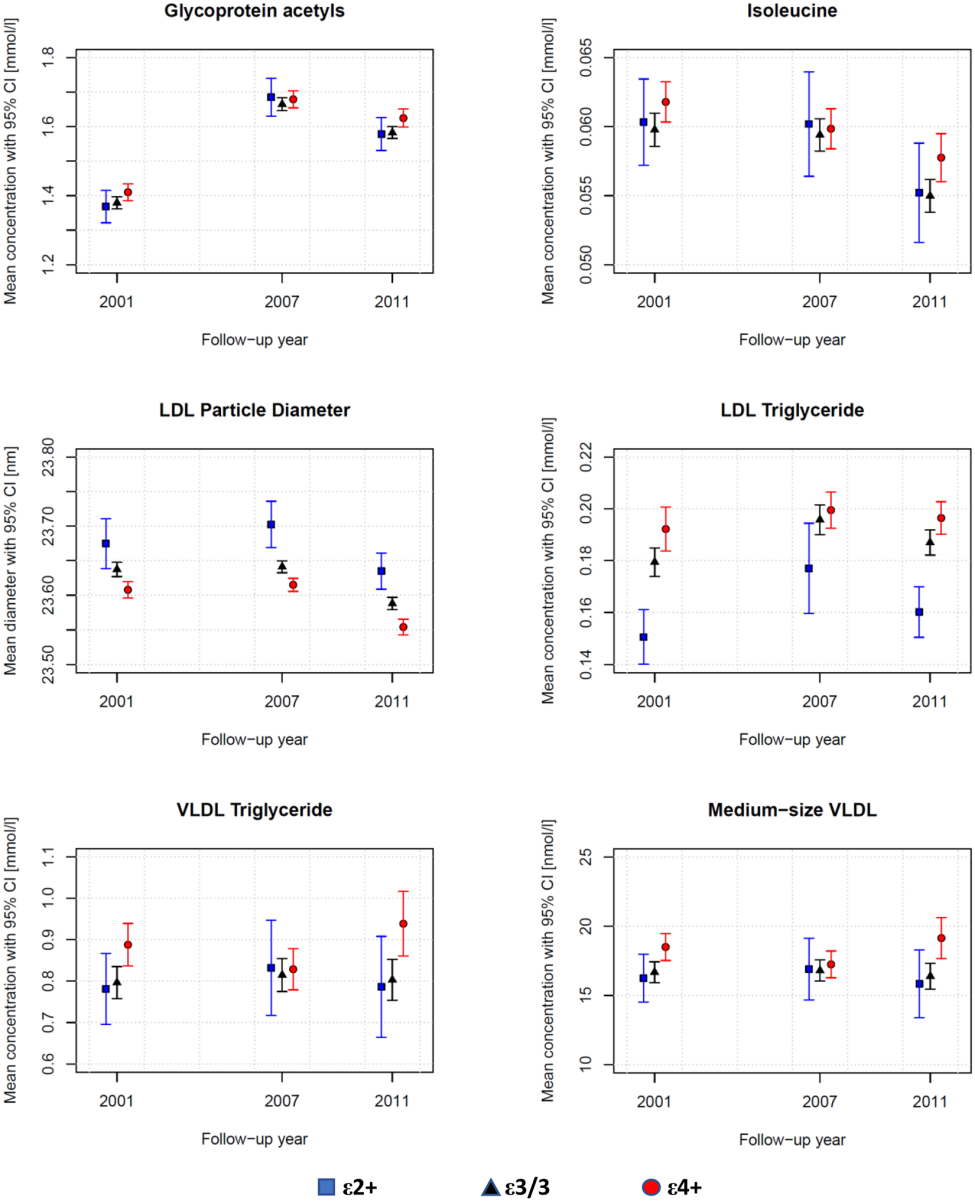
Repeated measures of six selected NMR-quantified and previously identified novel serum risk biomarkers in different apoE groups of all subjects (n = 1471) participated every three follow-ups (2001, 2007 and 2011) within YFS cohort. Statistics: According to two-way repeated measures analyses of variances, every presented metabolic measure expresses a main effect of apoE with p < 0.05 without any main effect of time or interaction effect (p ≫ 0.05). ApoE subgroup means are shown in absolute scale with 95% CI. Definitions: apoE ε2+ subgroup (ε2/2, ε3/2 combined; n = 98; squares), apoE ε3/3 subgroup (n = 846; triangles) and apoE ε4+ subgroup (ε4/2, ε4/3, ε4/4 combined; n = 527; circles).

In the cross-sectional analyses of all subjects, as novel findings apoE ε4 associated with altered levels of previously identified risk biomarkers, the repeated measures averages of which (with 95% CI) are also illustrated in Fig. 5. One missing GlycA measure in 2001 and 2007, as well as four isoleucine measures in 2001 and one in 2007 were mean value replaced. The concentrations of inflammation and all-cause mortality reflecting GlycA, as well as diabetogenic branched chain amino acid isoleucine were elevated in apoE ε4+ group. Furthermore, smaller LDL particle diameter as well as increased concentrations of LDL-TG, VLDL-TG and (medium-size, M)VLDL have been linked to elevated risk of CAD – all associated also with apoE ε4+ in our present analyses. According to verifying repeated measures analyses of variances, main effect of apoE with p < 0.05 was found for every parameter presented in Fig. 5. However, there were no main effects of time or interaction effects (apoE × time) present (p ≫ 0.05). All exact p-values are listed in Supplementary Dataset S14.

## Discussion

In the present study, apoE polymorphism associated consistently and as expected over the followed years with plasma concentrations of basic lipids i.e., total cholesterol, LDL-C and apoB (as widely reported in literature, including the previous YFS follow-up^23^). However, as new information apoE ε4 allele also presented a consistent atherogenic effect across all measured apoE- (chylomicron remnants, VLDL, IDL, HDL) and apoB-containing (VLDL-, IDL-, LDL) lipoproteins along with their subfractions, showing an influence on particle concentrations of free/esterified cholesterol, triglycerides, phospholipids and total lipid contents, as well as on LDL and VLDL particle sizes in serum. In addition, ε2 allele demonstrated an opposite, protective impact. Furthermore, to our knowledge as previously unreported findings, apoE ε4 also associated with altered levels of certain previously identified novel risk biomarkers of CAD, inflammation, diabetes and all-cause mortality.

Our cross-sectional results of apoE genotype effects on serum lipid and lipoprotein metabolites in all subjects are consistently in line with the metabolic mechanisms reviewed in detail in^6,32,33^. Briefly summarized, apoE polymorphism alters the hepatic binding, uptake, and catabolism of apoE- and apoB-containing lipoproteins, as well as intestinal cholesterol absorption, bile acid formation and endogenous cholesterol synthesis. In addition, after fat ingestion the shifting of apoE proteins between lipoproteins, as well as lipoprotein conversion and production rates are all suggested to depend on the apoE phenotypes. In ε4 carriers these mechanisms can lead to hypercholesterolemia and pro-atherogenic circulating lipoprotein-cholesterol distributions such as more cholesterol enriched chylomicron remnants and apoB-containing lipoproteins, as well as higher VLDL-C/HDL-C ratio^5,6,32^. More recently, ε4 has also been reported to unfavourably change the lipid composition and distribution in cell plasma membranes, as well as to associate with increased cytoplasmic lipids^34^. Elevated membranous and cytoplasmic cholesterol levels have been demonstrated with ε4, and increased membranous phospholipids, as well as cytoplasmic cholesteryl esters and TGs have been found in apoE deficiencies – all possibly promoting various heart anomalies^34^. The role of ε2 allele has been debated on one hand to be protective against dyslipidaemias and CAD, but on the other hand to promote type III hyperlipoproteinemia. However, the adverse effect of ε2 has been reported to manifest only in case of rare homozygosity (ε2/2) combined with additional genetic or environmental stress, e.g., obesity and high-fat diet^6^. On the other hand, references indicate possible race-dependence, e.g., ε2 being protective among Caucasians, but a potential risk factor in Asians^17^. In our Caucasian study population with only a few ε2/2 homozygotes ε2 has clearly a protective role – although, the relative quantities of TGs are elevated among the ε2+ subgroup (see ‘Lipid ratios’ in Figs 1–3). In addition, ε2 would seem to have even a bigger impact on our study population than ε4, which is well in line with the reference findings of ε2 cholesterol-lowering effect being 2–3 times that of the ε4 cholesterol-raising effect^5^.

In our longitudinal association analyses comparing metabolite level differences, a consistent increase of VLDL-related serum metabolites and TGs was found in the ε4+ subgroup (see Fig. 4). Results limited with FDR-corrected p < 0.05 were found only in the follow-up interval of 2007–2011 – however, as illustrated in Fig. 5 with VLDL-TG and MVLDL particle concentrations, this might be reasoned by variable metabolite trending along our follow-up period, experienced also in other reference studies such as in^35^ with serum cholesterols. Moreover, longitudinal associations were found only after adding the change of BMI into the multivariate linear regression model as a covariate – distinguishing the parallel effect of ε4 allele from the overall weight-gain throughout our entire study population (increase of mean BMI also notable in Table 1 and Supplementary Table S2). The number of reference studies reporting longitudinal follow-up findings is very limited. However, our results are well in line with an early study of standard serum lipid profile changes, where increased TG levels were found in the ε4+ subgroup in conjunction with a weight gain^36^. TG elevation was suggested to be caused by an increased VLDL production due to weight-gain, accompanied by retarded VLDL clearance attributable to the ε4 allele – the mechanism of which is explained deeper in a review by Phillips^6^. Indeed, our longitudinal, as well as cross-sectional results could well support this theory. Epidemiology and genetics have indicated that elevated TG-rich lipoproteins represent risk factors for low-grade inflammation, atherosclerotic CVD, and all-cause mortality^37^.

Low total HDL-C level, which has been epidemiologically linked to an increased risk of atherogenesis as a biomarker^38^, was at least partially evident in our ε4 carriers – observed at the end of our follow-up (2011) in all subjects. More consistently, ε4 allele associated with low cholesterol of large- and medium-sized HDL-particles. However, also ε2 carriers indicated lower cholesterol levels of extremely large and small HDL-particles, as well as of HDL3 subfractions. Based on present knowledge, it may be difficult to interpret whether the cholesterol contents of different HDL subpopulations could have any predictive value. Recent human genetics findings indicate that HDL-C concentration is unlikely a causative factor of atherosclerotic CVD^39,40^. In addition, a comprehensive literature review of HDL2 and HDL3 cholesterol does not distinguish cardioprotective differences between HDL subclasses, despite prior opposite hypothesis^41^. Furthermore, ongoing discussion and research have pointed out the extremely heterogenous composition of HDL particles with diverse functionalities, undergoing constantly dynamic changes of protein and lipid contents^38,42,43^. Hence, HDL-C may be a snapshot of a highly dynamic process, not describing as such the HDL particle structure or functionality. Nevertheless, certain variation of the HDL cholesterol profiles persisted between our apoE subgroups, assessed by multiple measurements.

Increasing evidence points towards evoked inflammatory mechanisms behind the early development of atherosclerotic plaque formation^44^. In terms of apoE ε4-promoted early inflammation, a previous YFS follow-up study discovered lower hs-CRP levels in certain subgroups of ε4 carriers – not giving evidence as such for this hypothesis^45^. On the other hand, glycoprotein acetyls (GlycA) is a novel biomarker of systemic inflammation, and elevated baseline circulating GlycA has been found to associate also with a future risk of CAD^14,46,47^. In our present cross-sectional analyses in the same YFS cohort, elevated GlycA level was found in the apoE ε4+ subgroup: In all subjects in the 2001 and 2011 follow-ups (Figs 1, 3 and 5), as well as in men separately in the 2011 follow-up (see Supplementary Figure S6). This new finding could support the role of apoE ε4 in possible early inflammatory provoked processes – either as a promoter, or a biomarker. In addition to inflammation, GlycA has also been reported to strongly predict the short-term risk of all-cause mortality^28,48^.

Small LDL particle diameter has been independently linked to atherogenesis and elevated risk of CVD, as well as all-cause mortality – potentially due to decreased LDL receptor binding and longer residence time in plasma, increased permeability through the endothelial barrier, arterial proteoglycan binding and susceptibility to oxidation (e.g.^49,50^). In our results, the difference in LDL diameter between the apoE subgroups persisted throughout the entire follow-up interval – ε4 allele associating with decreased, ε2 with increased mean diameter compared with ε3/3.

Increased levels of certain branched chain amino acids in the blood have been reported to flag up potentially developing diabetes mellitus type 2 later in life – possibly due to uncoupling of insulin receptors by persistent activation of mTOR pathway, or mitochondrial dysfunction by accumulation of toxic amino acid metabolites^51^. A recent review associate isoleucine (and leucine, not valine) with insulin resistance and higher blood glucose levels^51^. Isoleucine has been previously associated with insulin resistance also in the YFS cohort follow-up of 2001–2007^52^ – although this effect was only seen in men. In our current analyses, isoleucine was elevated by the ε4 allele in all subjects in 2001 and 2011 (see Figs 1 and 3 respectively), as well as in men in 2011 (see Supplementary Fig. S6).

As mentioned in the Introduction, LDL-C as such may not have the sensitivity or specificity needed to distinguish CAD risk within all the subjects with elevated LDL-C^14^. On the other hand, many CAD patients may have fairly normal LDL-C^15^. One of the suggested markers for predicting better the atherogenic potential of LDL is LDL-TG – high LDL-TGs being reported to indicate cholesterol ester-depleted LDL, elevated IDL and dense LDL^15^. In our analyses, lowered LDL-TG levels by the ε2 allele and elevated by the ε4 allele can be noted throughout the entire follow-up period. On the other hand, there is also discussion on turning focus from mere LDL also to IDL and VLDL – receiving support from the reviewed novel biomarkers for CAD, as well^14^. Higher concentrations of IDL, MVLDL and SVLDL have been associated with CAD risk, also in a Finnish population^53^ – all of which are elevated by the ε4 allele in our analyses. And as discussed earlier, our longitudinal analyses would indicate even a growing tendency of VLDL and TG over time in the ε4 carriers (see Fig. 4).

Our study has several important strengths. There are no prior systematic studies on the effect of apoE polymorphism on serum metabolome, most studies having limited to standard lipid panels. Moreover, earlier apoE studies have not implemented the NMR-method, which allows more detailed analyses and better resolution to compare different subgroups – especially important in distinguishing variations already prior to diagnosed disorders. In addition, our longitudinal follow-up with a continuously high participation rate revealed statistically consistent patterns evident within clusters of interlinked metabolic measures, significantly limiting the likelihood of mere isolated random positive hits. Also, the comprehensive background information gathered from our cohort allowed a thorough analysis and consideration of the most expected confounding factors. Furthermore, our analyses reflect genotype-related differences in a basically healthy general population – avoiding confounding/confusing expressions of illnesses expected when using morbid study subjects.

We also acknowledge certain limitations in our study. As previously reported in Finnish general population^7,13^, subgroups of apoE ε2/2, ε4/2, as well as ε4/4 were small also in our population-based setting and did not allow complete analyses of all the six genotypes separately. And although our cohort was not small by any means, its size may still have limited a completely comprehensive comparison between (sex-stratified) men and women, as more statistical power may be needed to distinguish all the small, emerging metabolic differences – nevertheless, the absence of apoE x sex interactions was confirmed in the analyses of all subjects. Finally, dietary fat intake, the rate of which was uncontrolled during our standard follow-up, has been reported to influence the magnitude of the observed exposure to apoE genotypes^54^, e.g., by altering the LDL-receptor binding of the E4 isoform^55^ – thus, generally lower levels of SFA intake within study subjects may possibly lead to smaller apoE genotype-related differences in serum metabolic measures. Nevertheless, based on reference studies (e.g.^5,7,54^) these recognized limitations do not alter the effect directions related to different apoE alleles, and are thus less likely to have a major impact on our main discoveries presented and discussed here.

In conclusion, based on a thorough NMR quantification of serum metabolome apoE polymorphism is associated with a complex metabolic change, including aberrations in multiple new biomarkers related to elevated cardiometabolic and all-cause mortality risk, extending our understanding about the role of apoE in health and disease. These changes are already evident in a basically healthy general population. For further work, the associations of more specifically quantified serum lipidomics data should also be analysed over the apoE genotypes, as certain TGs, cholesterol esters, PGs (phosphatidylethanolamines) and sphingomyelins (ceramides) have presented the strongest found novel associations with increased risk of CAD^14,56^ – in our study, elevated associations of all these lipid groups can be detected also in the apoE ε4+ subgroup (lowered in the ε2+ subgroup), and it is well expected to reveal more common nominators in the reported pool of novel risk markers. The relationship between the current new metabolic findings and pathogenesis, as well as different clinical manifestations should also be investigated using e.g., metabolic pathway analysis approach. For future analysis and upcoming end-points, the follow-up of the YFS cohort is also continuing.

## Electronic supplementary material

Supplementary Dataset 1

## Data Availability

The datasets generated and analysed during the current study are not publicly available due to legal reasons as the contained information could compromise research participant privacy and consent. For collaborative studies data can be inquired from the YFS coordinator (Prof. Olli Raitakari).
