# Supplementary material for: The effect of apolipoprotein E polymorphism on serum metabolome – a population-based 10-year follow-up study
Source: Sci Rep. 2019 Jan 24;9:458. doi: 10.1038/s41598-018-36450-9 (PMC6346097; doi:10.1038/s41598-018-36450-9)
Supplement: Supplementary file 1 — Supplementary Dataset 1 [file 41598_2018_36450_MOESM1_ESM.docx]

**Supplementary Material to**

**The effect of apolipoprotein E polymorphism on serum metabolome – a population-based 10-year follow-up study**

Juho-Pekka Karjalainen^1,*^, Nina Mononen^1^, Nina Hutri-Kähönen^2^, Miikael Lehtimäki^1^, Markus Juonala^3^, Mika Ala-Korpela^4^, Mika Kähönen^5^, Olli Raitakari^6^, Terho Lehtimäki^1^

^1^Department of Clinical Chemistry, Fimlab Laboratories and Finnish Cardiovascular Research Center-Tampere, Faculty of Medicine and Life Sciences, University of Tampere, Tampere, Finland.

^2^Department of Pediatrics, Tampere University Hospital and Faculty of Medicine and Life Sciences, University of Tampere, Finland.

^3^Department of Medicine, University of Turku, and Division of Medicine, Turku University Hospital, Turku, Finland, Murdoch Children’s Research Institute, Melbourne, Victoria, Australia. ^4^Computational Medicine, Faculty of Medicine, University of Oulu and Biocenter Oulu, Finland.

^4^NMR Metabolomics Laboratory, School of Pharmacy, University of Eastern Finland, Kuopio, Finland. ^4^Medical Research Council Integrative Epidemiology Unit at the University of Bristol, Bristol, UK. ^4^Population Health Science, Bristol Medical School, University of Bristol, Bristol, UK. ^4^Systems Epidemiology, Baker Heart and Diabetes Institute, Melbourne, VIC, Australia. ^4^Department of Epidemiology and Preventive Medicine, School of Public Health and Preventive Medicine, Faculty of Medicine, Nursing and Health Sciences, The Alfred Hospital, Monash University, Melbourne, VIC, Australia.

^5^Department of Clinical Physiology, Tampere University Hospital, and Finnish Cardiovascular Research Center - Tampere, Faculty of Medicine and Life Sciences, University of Tampere, Tampere, Finland.

^6^Department of Clinical Physiology and Nuclear Medicine, Turku University Hospital, and Research Centre of Applied and Preventive Cardiovascular Medicine, University of Turku, Turku, Finland.

*** Contact information for corresponding author:**

DSc, BM Juho-Pekka Karjalainen

Address: Faculty of Medicine and Life Sciences,

P.O. Box 100, FIN-33014 University of Tampere, FINLAND

Telephone: +358 50 582 7280

E-mail: juhopekka.karjalainen@gmail.com

**Content**

Supplementary Tables

Supplementary Table S1: *Description of the Metabolomics of YFS by Nuclear Magnetic*

*Resonance Spectroscopy* ………………………………………….......... 3

Supplementary Table S2: *Summary descriptive data for the YFS cohort …………*............................8

Supplementary Table S3: *Frequencies of different apoE genotypes in the YFS cohort* …………… 9

Supplementary Figures

Supplementary Figure S4: *Effects of apoE on NMR-based serum metabolic measures in the sex-*

*stratified YFS cohort in 2001*…………………………………………... 10

Supplementary Figure S5: *Effects of apoE on NMR-based serum metabolic measures in the sex-*

*stratified YFS cohort in 2007*……………………………………………11

Supplementary Figure S6: *Effects of apoE on NMR-based serum metabolic measures in the sex-*

*stratified YFS cohort in 2011*…………………………………………... 12

Supplementary Data

Supplementary Data S7: *Complete data with p-values from the cross-sectional association*

*analyses of all YFS subjects in 2001*.……………………….……………. 13

Supplementary Data S8*: Complete data with p-values from the cross-sectional association*

*analyses of all YFS subjects in 2007*………………………………........... 16

Supplementary Data S9: *Complete data with p-values from the cross-sectional association*

*analyses of all YFS subjects in 2011*.………………….…………………. 19

Supplementary Data S10: *Complete data with p-values from the cross-sectional association*

*analyses between YFS homozygotes ε3/3 and ε4/4 in 2001*.……………. 22

Supplementary Data S11: *Complete data with p-values from the cross-sectional association*

*analyses between YFS homozygotes ε3/3 and ε4/4 in 2007*.……………. 25

Supplementary Data S12: *Complete data with p-values from the cross-sectional association*

*analyses between YFS homozygotes ε3/3 and ε4/4 in 2011*.……………. 28

Supplementary Data S13: *Complete data with p-values from the longitudinal (level differences)*

*association analyses of all YFS subjects in 2007-2011*………………… 31

Supplementary Data S14: *Two-way repeated measures analyses of variances p-values*

*for the measured metabolites illustrated in Figure 5*…………….…...… 34

**Supplementary Table S1:** Description of the Metabolomics of YFS by Nuclear Magnetic Resonance Spectroscopy

The lipoprotein subclasses were defined based on high-performance liquid chromatography as follows:

**XXL** **VLDL** (*chylomicrons and extremely large VLDL,* particle diameters from approx. 75 nm upwards)

**XL** **VLDL** (*very large*, average particle diameter 64.0 nm)

**L** **VLDL** (*large*, 53.6 nm); **M** **VLDL** (*medium*, 44.5 nm); **S** **VLDL** (*small*, 36.8 nm); **XS** **VLDL** (*very small*, 31.3 nm)

**IDL** (28.6 nm); **L** **LDL** (*large*, 25.5 nm); **M LDL** (*medium*, 23.0 nm); **S LDL** (*small*, 18.7 nm)

**XL HDL** (*very large*, 14.3 nm); **L HDL** (*large*, 12.1 nm); **M HDL** (*medium*, 10.9 nm); **S HDL** (*small*, 8.7 nm)

**Supplementary Table S2:** Summary descriptive data for the YFS cohort in 2001, 2007 and 2011. Values are mean (SD) or n (%).

|  |  | **2001** |  |  | **2007** |  |  | **2011** |  |
| --- | --- | --- | --- | --- | --- | --- | --- | --- | --- |
|  | **All** | **Male** | **Female** | **All** | **Male** | **Female** | **All** | **Male** | **Female** |
| Number of subjects | 2234 | 1004 (44.9) | 1230 (55.1) | 2148 | 970 (45.2) | 1178 (54.8) | 1918 | 860 (44.8) | 1058 (55.2) |
| Age [years] | 31.7 (5.0) | 31.7 (5.0) | 31.7 (5.0) | 37.6 (5.0) | 37.6 (5.0) | 37.7 (5.0) | 41.9 (5.0) | 41.9 (5.1) | 41.9 (5.0) |
| BMI [kg/m²] | 25.1 (4.4) | 25.7 (4.1) | 24.5 (4.6) | 26.0 (4.8) | 26.8 (4.3) | 25.4 (5.1) | 26.5 (5.0) | 27.0 (4.3) | 26.1 (5.5) |
| Daily smokers | 533 (23.9) | 296 (29.5) | 237 (19.3) | 395 (18.4) | 219 (22.6) | 176 (14.9) | 263 (13.7) | 133 (15.5) | 130 (12.3) |
| Cholesterol lowering medicated | 7 (0.3) | 6 (0.6) | 1 (0.1) | 45 (2.1) | 31 (3.2) | 14 (1.2) | 70 (3.6) | 48 (5.6) | 22 (2.1) |
| Diabetes mellitus type 2 | 1 (0.0) | 1 (0.1) | 0 (0.0) | 23 (1.1) | 13 (1.3) | 10 (0.8) | 69 (3.6) | 32 (3.7) | 37 (3.5) |
| Hypertension | 40 (1.8) | 22 (2.2) | 18 (1.5) | 122 (5.7) | 65 (6.7) | 57 (4.8) | 160 (8.3) | 81 (9.4) | 79 (7.5) |
|  |  |  |  |  |  |  |  |  |  |
| Total cholesterol [mmol/L] | 5.20 (1.12) | 5.16 (1.06) | 5.24 (1.17) | 5.31 (1.12) | 5.35 (1.13) | 5.27 (1.12) | 5.33 (1.07) | 5.40 (1.12) | 5.27 (1.02) |
| VLDL cholesterol [mmol/L] | 0.81 (0.31) | 0.90 (0.32) | 0.75 (0.29) | 0.81 (0.31) | 0.91 (0.33) | 0.73 (0.26) | 0.76 (0.33) | 0.88 (0.35) | 0.67 (0.27) |
| IDL cholesterol [mmol/L] | 0.83 (0.23) | 0.84 (0.22) | 0.82 (0.23) | 0.85 (0.22) | 0.87 (0.22) | 0.83 (0.21) | 0.86 (0.22) | 0.88 (0.23) | 0.83 (0.20) |
| LDL cholesterol [mmol/L] | 1.94 (0.60) | 2.00 (0.61) | 1.89 (0.60) | 1.99 (0.60) | 2.10 (0.61) | 1.91 (0.58) | 2.05 (0.60) | 2.17 (0.63) | 1.96 (0.57) |
| HDL cholesterol [mmol/L] | 1.78 (0.42) | 1.43 (0.33) | 1.78 (0.42) | 1.65 (0.43) | 1.47 (0.36) | 1.80 (0.44) | 1.65 (0.42) | 1.46 (0.36) | 1.81 (0.40) |
| Free cholesterol [mmol/L] | 1.50 (0.32) | 1.48 (0.30) | 1.52 (0.33) | 1.63 (0.32) | 1.64 (0.32) | 1.62 (0.32) | 1.61 (0.31) | 1.64 (0.32) | 1.60 (0.30) |
| Esterified cholesterol [mmol/L] | 3.70 (0.80) | 3.68 (0.77) | 3.72 (0.82) | 3.68 (0.81) | 3.71 (0.82) | 3.65 (0.81) | 3.71 (0.77) | 3.76 (0.81) | 3.68 (0.74) |
| Remnant cholesterol [mmol/L] | 1.64 (0.49) | 1.73 (0.49) | 1.57 (0.48) | 1.66 (0.47) | 1.78 (0.49) | 1.56 (0.44) | 1.62 (0.47) | 1.77 (0.49) | 1.50 (0.42) |
| Triglycerides [mmol/L] | 1.29 (0.69) | 1.45 (0.73) | 1.17 (0.62) | 1.31 (0.72) | 1.51 (0.79) | 1.14 (0.60) | 1.31 (0.87) | 1.57 (0.96) | 1.10 (0.73) |
| Total fatty acids [mmol/L] | 13.08 (3.01) | 13.09 (2.91) | 13.08 (3.08) | 13.74 (3.16) | 14.09 (3.22) | 13.45 (3.08) | 13.33 (3.34) | 13.77 (3.68) | 12.97 (2.99) |
| apoA-I [g/L] | 1.68 (0.27) | 1.59 (0.21) | 1.76 (0.28) | 1.71 (0.28) | 1.63 (0.23) | 1.77 (0.29) | 1.70 (0.24) | 1.62 (0.21) | 1.77 (0.25) |
| apoB [g/L] | 0.98 (0.24) | 1.03 (0.24) | 0.94 (0.23) | 0.99 (0.24) | 1.06 (0.24) | 0.94 (0.22) | 1.00 (0.25) | 1.08 (0.26) | 0.93 (0.22) |
| VLDL particle size [nm] | 36.31 (1.34) | 36.77 (1.41) | 35.94 (1.15) | 36.13 (1.37) | 36.61 (1.40) | 35.74 (1.20) | 36.12 (1.59) | 36.72 (1.66) | 35.64 (1.36) |
| LDL particle size [nm] | 23.63 (0.16) | 23.60 (0.16) | 23.66 (0.15) | 23.64 (0.13) | 23.60 (0.12) | 23.67 (0.13) | 23.58 (0.13) | 23.54 (0.13) | 23.61 (0.13) |
| HDL particle size [nm] | 9.94 (0.27) | 9.80 (0.24) | 10.06 (0.24) | 9.96 (0.27) | 9.82 (0.22) | 10.08 (0.25) | 9.95 (0.29) | 9.80 (0.24) | 10.08 (0.26) |

**Supplementary Table S3.** Frequencies, n (%), of different apoE genotypes in the YFS cohort in 2001, 2007 and 2011.

|  |  | **2001** |  |  | **2007** |  |  | **2011** |  |
| --- | --- | --- | --- | --- | --- | --- | --- | --- | --- |
|  | **All** | **Male** | **Female** | **All** | **Male** | **Female** | **All** | **Male** | **Female** |
| **ε2/2** | 4 (0.2) | 2 (0.2) | 2 (0.2) | 4 (0.2) | 2 (0.2) | 2 (0.2) | 4 (0.2) | 2 (0.2) | 2 (0.2) |
| **ε3/2** | 142 (6.4) | 46 (4.6) | 96 (7.8) | 140 (6.5) | 55 (5.7) | 85 (7.2) | 130 (6.8) | 47 (5.5) | 83 (7.8) |
| **ε3/3** | 1280 (57.3) | 585 (58.3) | 695 (56.5) | 1235 (57.5) | 566 (58.4) | 669 (56.8) | 1096 (57.1) | 495 (57.6) | 601 (56.8) |
| **ε4/2** | 44 (2.0) | 18 (1.8) | 26 (2.1) | 42 (2.0) | 17 (1.8) | 25 (2.1) | 36 (1.9) | 13 (1.5) | 23 (2.2) |
| **ε4/3** | 684 (30.6) | 317 (31.6) | 367 (29.8) | 646 (30.1) | 296 (30.5) | 350 (29.7) | 578 (30.1) | 271 (31.5) | 307 (29.0) |
| **ε4/4** | 80 (3.6) | 36 (3.6) | 44 (3.6) | 81 (3.8) | 34 (3.5) | 47 (4.0) | 74 (3.9) | 32 (3.7) | 42 (4.0) |
| **TOTAL** | 2234 | 1004 | 1230 | 2148 | 970 | 1178 | 1918 | 860 | 1058 |

**Supplementary Figure S4.** ApoE effects on 149/228 (p<0.05 after false discovery rate correction) NMR-based serum metabolic measures in men (n=1004) and women (n=1230) of YFS cohort participated in 2001. **Statistics:** Regression models are adjusted for age and BMI. Regression β-coefficients (x-axis) indicate in standard deviation (SD) units the change in metabolite level over apoE genotype subgroups (ε2+, ε3/3, ε4+). The most common ε3/3 subgroup (n=585 for men, n=695 for women) is set at the origin (zero SD) and post-hoc compared with ε2+ (squares, men blank and women filled) and ε4+ subgroups (circles, men blank and women filled). β-values with 95% CI are scaled to SD increments from normalized i.e., ln-transformed metabolic measures. For clarity of illustration, only the results of the analyses with p<0.05 after false discovery rate correction are shown here. **Definitions:** apoE ε2+ subgroup (ε2/2, ε3/2 combined; n=48 for men, 98 for women) and apoE ε4+ subgroup (ε4/2, ε4/3, ε4/4 combined; n=371 for men, 437 for women).

**Supplementary Figure S5.** ApoE effects on 104/228 (p<0.05 after false discovery rate correction) NMR-based serum metabolic measures in men (n=970) and women (n=1178) of YFS cohort participated in 2007. **Statistics:** Regression models are adjusted for age and BMI. Regression β-coefficients (x-axis) indicate in standard deviation (SD) units the change in metabolite level over apoE genotype subgroups (ε2+, ε3/3, ε4+). The most common ε3/3 subgroup (n=566 for men, n=669 for women) is set at the origin (zero SD) and post-hoc compared with ε2+ (squares, men blank and women filled) and ε4+ subgroups (circles, men blank and women filled). β-values with 95% CI are scaled to SD increments from normalized i.e., ln-transformed metabolic measures. For clarity of illustration, only the results of the analyses with p<0.05 after false discovery rate correction are shown here. **Definitions:** apoE ε2+ subgroup (ε2/2, ε3/2 combined; n=57 for men, 87 for women) and apoE ε4+ subgroup (ε4/2, ε4/3, ε4/4 combined; n=347 for men, 422 for women).

**Supplementary Figure S6.** ApoE effects on 153/228 (p<0.05 after false discovery rate correction) NMR-based serum metabolic measures in men (n=860) and women (n=1058) of YFS cohort participated in 2011. **Statistics:** Regression models are adjusted for age and BMI. Regression β-coefficients (x-axis) indicate in standard deviation (SD) units the change in metabolite level over apoE genotype subgroups (ε2+, ε3/3, ε4+). The most common ε3/3 subgroup (n=495 for men, n=601 for women) is set at the origin (zero SD) and post-hoc compared with ε2+ (squares, men blank and women filled) and ε4+ subgroups (circles, men blank and women filled). β-values with 95% CI are scaled to SD increments from normalized i.e., ln-transformed metabolic measures. For clarity of illustration, only the results of the analyses with p<0.05 after false discovery rate correction are shown here. **Definitions:** apoE ε2+ subgroup (ε2/2, ε3/2 combined; n=49 for men, 85 for women) and apoE ε4+ subgroup (ε4/2, ε4/3, ε4/4 combined; n=316 for men, 372 for women).

**Supplementary Data S7:** Complete data including all p-values from the cross-sectional association analyses of all YFS subjects in 2001. **Annotation:** FDR p/apoE, false discovery rate corrected F-test p-value for apoE; Beta, post-hoc β-coefficient; ci.low, 95% post-hoc CI lower limit; ci.high, 95% post-hoc CI upper limit; FDR p/ε2+ or ε4+, false discovery rate corrected post-hoc p-value.

**Supplementary Data S8:** Complete data including all p-values from the cross-sectional association analyses of all YFS subjects in 2007. **Annotation:** FDR p/apoE, false discovery rate corrected F-test p-value for apoE; Beta, post-hoc β-coefficient; ci.low, 95% post-hoc CI lower limit; ci.high, 95% post-hoc CI upper limit; FDR p/ε2+ or ε4+, false discovery rate corrected post-hoc p-value.

**Supplementary Data S9:** Complete data including all p-values from the cross-sectional association analyses of all YFS subjects in 2011. **Annotation:** FDR p/apoE, false discovery rate corrected F-test p-value for apoE; Beta, post-hoc β-coefficient; ci.low, 95% post-hoc CI lower limit; ci.high, 95% post-hoc CI upper limit; FDR p/ε2+ or ε4+, false discovery rate corrected post-hoc p-value.

**Supplementary Data S10:** Complete data with p-values from the cross-sectional association analyses between YFS homozygotes ε3/3 (n=1280) and ε4/4 (n=80) in 2001. **Annotation:** Beta ε4/4, (post-hoc) β-coefficient; ci.low, 95% ε4/4 (post-hoc) CI lower limit; ci.high ε4/4, 95% (post-hoc) CI upper limit; FDR p ε4/4, false discovery rate corrected (post-hoc) p-value.

**Supplementary Data S11:** Complete data with p-values from the cross-sectional association analyses between YFS homozygotes ε3/3 (n=1235) and ε4/4 (n=81) in 2007. **Annotation:** Beta ε4/4, (post-hoc) β-coefficient; ci.low, 95% ε4/4 (post-hoc) CI lower limit; ci.high ε4/4, 95% (post-hoc) CI upper limit; FDR p ε4/4, false discovery rate corrected (post-hoc) p-value.

**Supplementary Data S12:** Complete data with p-values from the cross-sectional association analyses between YFS homozygotes ε3/3 (n=1096) and ε4/4 (n=74) in 2011. **Annotation:** Beta ε4/4, (post-hoc) β-coefficient; ci.low, 95% ε4/4 (post-hoc) CI lower limit; ci.high ε4/4, 95% (post-hoc) CI upper limit; FDR p ε4/4, false discovery rate corrected (post-hoc) p-value.

**Supplementary Data S13:** Complete data including all p-values from the longitudinal (level differences) association analyses of all YFS subjects in 2007-2011. **Annotation:** Beta, (post-hoc) β-coefficient; ci.low, 95% (post-hoc) CI lower limit; ci.high, 95% (post-hoc) CI upper limit; FDR p/ε4+, false discovery rate corrected (post-hoc) p-value.

**Supplementary Data S14:** Two-way repeated measures analyses of variances p-values for the measured metabolites illustrated in Figure 5. **Annotation:** p/apoE, apoE genotype main effect p-value; p/time, time main effect p-value; p/interaction, apoE x time interaction p-value.

| **Metabolite** | **p/ apoE** | **p/ time** | **p/ interaction** |
| --- | --- | --- | --- |
| **GlycA** | 3.56E-03 | 6.22E-01 | 3.50E-01 |
| **Isoleucine** | 2.21E-02 | 3.75E-01 | 6.20E-01 |
| **LDL diameter** | 1.88E-06 | 7.78E-01 | 1.71E-01 |
| **LDL-TG** | 7.02E-05 | 6.82E-01 | 8.94E-01 |
| **VLDL-TG** | 4.81E-03 | 9.12E-01 | 5.89E-01 |
| **MVLDL** | 9.77E-04 | 9.28E-01 | 5.50E-01 |
